# *N*-acetylgalactosaminyltransferases in cancer

**DOI:** 10.18632/oncotarget.10042

**Published:** 2016-06-14

**Authors:** Muhammad Ramzan Manwar Hussain, Daniel C. Hoessli, Min Fang

**Affiliations:** ^1^ CAS Key Laboratory of Pathogenic Microbiology and Immunology, Institute of Microbiology, Chinese Academy of Sciences, Beijing, China; ^2^ University of Chinese Academy of Sciences, Beijing, China; ^3^ Dr. Panjwani Center for Molecular Medicine and Drug Research, International Center for Chemical and Biological Sciences, University of Karachi, Karachi, Pakistan

**Keywords:** N-acetylgalactosaminyltransferases, O-linked glycoprotein biosynthesis, cancer markers

## Abstract

Aberrant mucin-type O-glycosylation by glycosyltransferases is a well-described hallmark of many cancers and is also associated with additional non-cancerous developmental and metabolic disorders. The current review focuses on *N*-acetylgalactosaminyltransferase genes (*GALNT*s) and proteins (GalNAcTs) to illustrate their importance in cancer biology. Aberrant O-glycosylation by GalNAcTs activates a wide range of proteins that carry out interactions of sessile and motile cells affecting organogenesis, responses to agonists and stimulating hyperproliferation and metastatisation of neoplastic cells. As genome-wide analyses have provided abundant clues regarding under- or over-expressed genes that characterize different types of cancers, *GALNTs* and their transferase products have attracted attention by being unexpected actors in neoplastic contexts. We intend to review the current knowledge on *GALNTs* and their encoded transferases in cancer and suggest what could be the significance of such information in cancer pathogenesis and management.

## INTRODUCTION

In many aspects of development and disease, critical genetic and epigenetic alterations of genes encoding glycosyltansferases can cause pathologic changes [1-4]. *N*-linked and *O*-linked sugars are the two major glycosylation forms observed in secreted and cell-surface proteins. *N*-linked sugars contribute to protein folding, secretion and stability. Endoplasmic reticulum stress responses and cellular apoptosis are activated when *N*-glycosylation is disrupted. *O*-glycosylated proteins are also found in cell surface, serum and in the extracellular matrix (ECM). Therefore, altered cell-surface *O*-glycoproteins are often implicated in uncontrolled proliferation, invasion and metastasis [5], and similarly, the *O*-glycosylated ECM proteins are often involved in a variety of developmental pathologies. The intracellular localization of *N*-acetylgalactosaminyltransferases may become altered in cancer cells by redistributing to the endoplasmic reticulum, rather than being restricted to the Golgi [6, 7]. Alterations in protein O-glycosylation by a family of over 20 polypeptide GalNAc-transferases (GalNAcTs or GALNTs) catalyzing the transfer of *N*-acetylgalactosamine (GalNAc) from UDP-GalNAc to the hydroxyl group of a serine or threonine residue have been linked with a wide variety of epithelial developmental defects and neoplasms [4-6, 8-10]. The unusually large number of GalNAcTs is unique to O-glycosylation and the multiplicity of conserved isoforms in metazoan evolution suggests a need for cell or tissue-specific isoforms [11].

The GalNAcTs generate Tn antigens by transferring αGalNAc from UDP-GalNAc to Ser and Thr residues [12]. This Tn antigen may become sialylated on C6 to form sialyl-Tn antigen, or galactosylated on C3 to form the T antigen (or TF: Thomsen-Friedenreich, antigen) [12].

Studies on domain organizations of the different GalNAcTs have provided essential information on their enzymatic functions [13]. The type II transmembrane GalNAcTs insert into the Golgi membranes *via* a non-cleaved membrane-spanning domains and are connected with a luminal catalytic domain by a stem region. The catalytic domain of GalNAcTs is linked to a C-terminal, ricin-like lectin domain, a unique feature of eukaryotic GalNAcTs'. The linker sequence between catalytic and lectin domains is also important for the lectin domain to assist in the catalytic activity of the enzyme, but distinct GalNAc glycopeptide binding properties of different GalNAcTs lectin domains suggest additional functions beyond the regulation of catalytic domains [11, 13, 14].

GalNAc-type *O*-glycosylation may also correlate with proprotein convertase processing of >3000 protein substrates [15]. For instance, O-glycosylation in the vicinity of preprocessing sites have been shown to be altered in the rare disease familial tumoral calcinosis [16]. Using synthetic glycopeptides, Schjoldlager et al. 2011 have shown that *O*-glycans located +/- 3 residues from the R*XX*R furin cleavage site exert a co-regulatory role in preprotein processing [15].

Expression of *GALNTs* and their encoded GalNAcT proteins occurs in a wide range of tissues [6, 7, 17-19]. The distinct functions of their domains in the glycosylation process itself and their preferences for peptides or glycopeptides may contribute to a wide variety of disease-risks [4, 11, 13, 18, 20-24]. This review intends to highlight the neoplastic contexts where *GALNTs* are expressed and show their potential clinical usefulness for diagnostic and prognostic purposes.

### *N*-Acetylgalactosaminyltransferase 1

Aberrant glycosylation resulting from mutations in *GALNT1* was found to be involved in cardiac defects, and in melanoma, ovarian and bladder cancers [9, 11, 21, 23, 25, 26].

Expression of *GALNT1* is required for O-glycosylation of many proteins of the extracellular matrix and basement membrane that regulate the normal heart valve development and submandibular gland development. Consequently, the loss of *GALNT1* increases the bone morphogenetic protein (BMP) kinase and mitogen-activated protein kinase (MAPK) signaling, which are major contributing factors to the multiplication of endocardial cushion cells and integrin signaling in submandibular epithelial cells [9]. This phenotype suggests that transmembrane serine/threonine kinases are functionally altered in the absence GalNAcT1 and points to the incapacity of the TGFβ-family receptors to link the message of the agonist (BMP) to intracellular downstream signaling pathways (MAPK) [9].

In hepatocellular carcinoma (HCC), GalNAcT1 is frequently upregulated, which facilitates HCC cell migration and invasion by increasing the O-glycan addition to EGFR. Reciprocally, downregulation of GalNAcT1 decreases EGFR O-glycosylation and reduces the malignant behavior of HCC by decreasing EGF-stimulated EGFR phosphorylation. Hypophosphorylated EGFR is then internalized, which terminates EGF-induced signaling. In addition to EGF, downregulation of GalNAcT1 also decreases the PDGF- and VEGF-induced invasion of HCC [27].

In A375 human melanoma cells exposed to kojic acid (a tyrosinase inhibitor and skin whitener), seven likely tumor-suppressor genes— *GALNT1, APOBEC1, ARHGEF16, CD22, FGFR3, UNC5C* and *ZNF146—* were shown to be down-regulated, resulting in the loss of suppressor gene function [26]. The single-nucleotide polymorphism rs17647532 in *GALNT1* was genotyped in fourteen studies to suggest association between *GALNT1* alterations and ovarian cancer, but could not be confirmed in large populations [21, 25, 28]. In bladder cancer cells, expression of *GALNT1*-mRNA was higher by eleven-fold compared to normal cells, suggesting that hyperexpressed *GALNT1* could be a novel marker for human bladder cancer [22]. In another report, a direct linkage between miR-129 and its putative targets *SOX4* and *GALNT1* have opened the possibility of a differential regulation of such genes in bladder cancer [23].

### *N*-Acetylgalactosaminyltransferase 2

Different genome-wide association studies (GWAS) have documented the association of common variants of *GALNT2* with both high-density lipoprotein cholesterol (HDLc) and triglyceride levels [24, 29]. A GWAS study identified the functional expression of *GALNT2*, *TRIB1* and *SORT1* in association with changes in lipid levels and degrees of heart disease [30]. Of the six newly identified chromosomal regions, one was associated with HDL cholesterol, two with low-density lipoprotein (LDL) cholesterol and five with triglycerides have been correlated with risk factors for cardiovascular disease [10]. Additionally, in mouse models, knockdown and overexpression of *GALNT2* were inversely related to HDLc levels. Owing to the high homology between mouse and human transferase genes, the linkage of three newly identified human genes (*GALNT2, WWOX*, and *CDH13*) were suggested *in silico* to be associated with HDL levels [31].

*GALNT2* overexpression altered the Tn antigen expression by neuroblastoma (NB) cells and suppressed malignant proliferation of NB cells, as a result of decreased dimerization of IGF-1R (Insulin-like growth factor receptor), a critical glycosylation target for GalNAcT2 [32]. The activation of IGF-1Rs following *GALNT2* knockout suggests that non-glycosylated IGF-1R dimerizes and increases the IGF-l (Insulin-like growth factor)-induced signals for cancer cell growth, migration, and invasion. Reciprocally, over-expression of *GALNT2* significantly inhibited IGF-l-stimulated growth, migration, and invasion of NB cells, suggesting that *O*-glycosylated IGF-1R did not dimerize and thus failed to support the malignant behavior of NB cells [32]. Quite differently, in oral squamous cell carcinoma (OSCC), GalNAcT2 enhanced the invasive properties of OSCC cells by *O*-glycosylation the EGFR, and was selectively demonstrable in cells of the invading front of the tumor. In gastric adenocarcinoma, downregulation of GALNT2 increased the cell proliferation, migration, invasion and tumor metastasis by increasing the phosphorylation of hepatocyte growth factor receptor (MET: a receptor tyrosine kinase like IGF-l) and decreasing the expression of the Tn antigen on MET [33]. Likewise, downregulation of GalNAcT2 in hepatocellular carcinoma (HCC) cells promoted cell growth, migration, and invasion *in vitro* and *in vivo* by modulating the *O*-glycan pattern on EGFR, resulting in EGFR activation by dimerization, and phosphorylation of downstream signaling proteins [34]. In breast cancer cells (MDA231), increased Tn antigen amounts were observed in the endoplasmic reticulum (ER) concomitantly with GalNAcT2, thereby suggesting a role for COPI-based translocation of GalNAcT2 to the ER in increasing Tn levels in the ER [7]. In tumors such as breast, OSCC, HCC, and gastric carcinoma, future development of specific inhibitors of GalNAcTs (and GalNAcT2 in particular) might have potential therapeutic use [35].

Expression of *GALNT1, GALNT2, GALNT3, GALNT4, GALNT5, GALNT6, GALNT9* and *GALNT14* in B-cells has highlighted the significant role of *GALNTs* in B-cell biology. In particular, *GALNT2* activation was found necessary for the initiation of *O*- glycosylation of the IgA1 hinge region, but the lack of *GALNT1* resulted in decreased immunoglobulin G production, increased germinal center B-cell apoptosis and reduced numbers of plasma cells [36]. In myeloid cells, the expression of *GALNT2, GALNT4, GALNT5* and *GALNT7* was observed in K562 cells (chronic myeloid leukemia line), and of *GALNT1, GALNT2, GALNT3* and *GALNT4* in SHI-1 (acute monocytic leukemia line) [36, 37].

### *N*-acetylgalactosaminyltransferase 3

**Figure 1:**
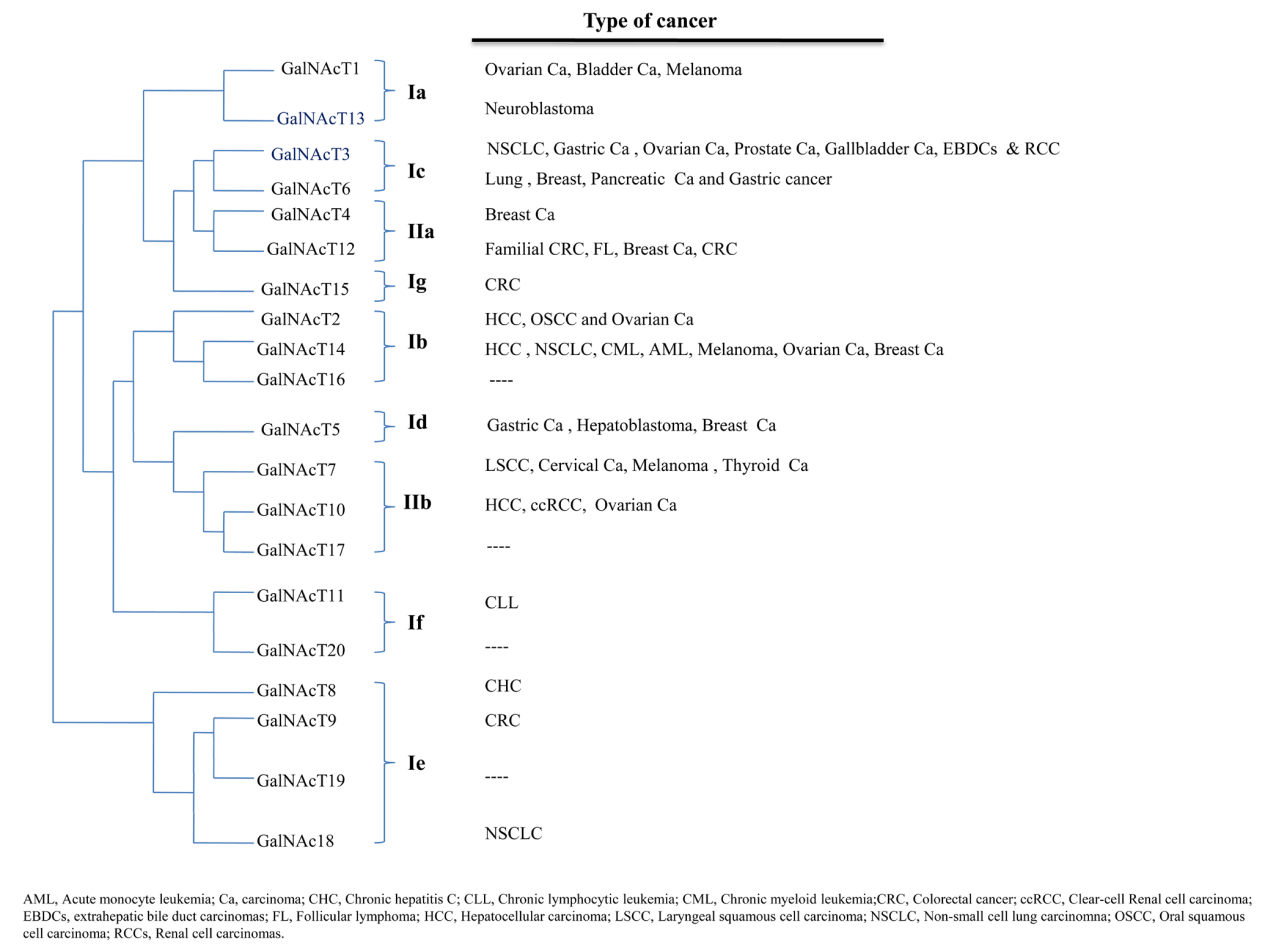
Phylogenetic tree showing the classification of GalNAcTs [11] The implication of each transferase in individual cancers is shown in relationship to the phylogenetic tree.

Owing to the high structural similarity between *GALNT3* and *GALNT6*, and the similar enzymatic properties of their encoded products, Bennett et al. proposed a classification of the twenty *GALNT* genes into distinct gene families [11, 38]. In this classification, the structurally identical *GALNT3* and *GALNT6* with nine intron/exon boundaries have been grouped in the Ic subfamily [11]. Except for *GALNT3* and *GALNT6*, no correlation was found between structure and co-expression of other transferases, either in the same cancer cell type, or in other specific clinicopathological contexts (Figure 1). Despite their structural similarities, *GALNT3* and *GALNT6* do not co-localize to a single locus, are differentially expressed (as in the WI38 fibroblast cell line) and glycosylate fibronectin differently [38, 39]. For instance, the GalNAcT6 enzyme, unlike GalNAcT3, *O*-glycosylates oncofetal fibronectin efficiently [38]. Likewise, GalNAcT6 was found to function as an independent prognostic marker in pancreatic cancer, although both GalNAcT3 and GalNAcT6 were co-expressed in a cohort of pancreatic cancer cases [40]. In another study of renal cell carcinomas (RCCs), GalNAcT3 was predicted as an independent prognostic factor for high-grade tumor and poor prognosis in RCC patients, whereas GalNAcT6 was not [41]. Finally, much more is needed to correlate GalNAcT3 and GalNAcT6 expression with their capacity to aberrantly O-glycosylate the Muc1, and how this could affect the morphology, invasiveness and migration properties of RCC [41, 42].

Mutations in *GALNT3* (c.484 C>T; p.R162X in exon1; c.1524+5G>A; splicing error in intron 7) impair the structure and function of GalNAcT3 and may cause modifications in growth regulation, immune recognition, and cell adhesion of cancer cells. Skipping of *GALNT3* exon 7 caused an in-frame deletion of 44 amino acids, eliminating the linker region between the catalytic and lectin domains of GalNAcT3 [43]. Previous studies of GalNAcT3 glycosylating glycopeptide substrates have suggested that the lectin domain is involved in binding glycopeptide substrates [13]. The glycosylation of the endocrine FGF23 (fibroblast growth factor 23) by GalNAcT3 occurs *via* a lectin-dependent, selective O-glycosylation at Thr178. This site is adjacent to the proprotein convertase (PC) cleavage site of FGF23, such that O-glycosylation prevents FGF23 proteolytic degradation. The loss of active GalNAcT3 results in excessive FGF23 proteolytic processing and FGF23 deficit. However, prior to FGF23 Thr178 glycosylation, the first glycosylation occurs at the Thr171 in a lectin-independent manner and may serve as a target switching or enhancer for the second glycosylation at Thr178 [13, 16, 43].

The low expression of *GALNT3* in non-small cell lung carcinoma (NSCLC) was shown to be an unfavorable prognostic factor for stage-I NSCLC and stage-I non-squamous cell carcinomas [44]. Altered GalNAcT3 expression may decrease protein O-glycosylation, resulting in altered biological activities of NSCLCs and shorter survival times [44-47]. Strong GalNAcT3 expression was characterized in both early-stage undifferentiated and differentiated gastric carcinomas whereas higher frequencies of metastatic lymph node involvement was seen in undifferentiated gastric carcinomas [48]. In addition, GalNAcT3 expression was correlated with the aggressive behavior of oesophageal squamous cell carcinomas [49]. In a recent report on papillary thyroid carcinoma (PTC), *WNK1*-*B4GALNT3* fusion was correlated with the overexpression of *B4GALNT3* [50]. In another study, GalNAcT3 expression in gastric carcinoma was found to positively correlate with differentiation. High expression of GalNAcT3 was more frequent in differentiated carcinomas and low expression of GalNAcT3 in less differentiated, more malignant carcinomas [11, 51].

Similarly, iprostate*in situ* In a study of extrahepatic bile duct carcinomas (EBDCs), GalNAcT3 expression during tumor growth was found associated with lymph node metastasis [54]. Likewise, GalNAcT3 expression in RCC patients correlated with RCC invasion and metastasis, probably by decreasing O-glycosylation on cell-adhesion molecular markers *β*-catenin and E-cadherin and decreasing tumor cell adhesion to the stroma [41]. However, in other studies, weak GalNAcT3 expression was associated with poor differentiation and aggressiveness of ductal adenocarcinoma of the pancreas [41, 55].

### *N*-acetylgalactosaminyltransferase 4

N-acetylgalactosaminyltransferase 4 (*GALNT4*) encoding the fourth human N-acetylgalactosaminyltransferase (GalNAcT4) shares GalNAc-glycopeptide substrates with GalNAcT12, and was classified as group IIa [11].

Estrogens and estrogen receptors (ERs) are critical regulators of breast cell tumorigenesis. By quantitative, real-time PCR (qPCR), five genes (SYTL5, RAB27B, SNX24, GALNT4 and SLC12A2/NKCC1/BSC2) have been implicated in estrogen regulation [56]. How does the vesicle trafficking genes SYTL5, RAB27B, SNX24 and SLC12A2 cooperate with GALNT4 to mediate the 17β-estradiol (E2) signaling in breast cancer cells is another key question. Immunohistochemical detection of GalNAcT4 in renal tumor cells of relapse-free surviving patients with clear-cell renal cell carcinoma (ccRCC) strongly suggested that GALNT4 expression is a positive prognostic factor [57].

### *N*-acetylgalactosaminyltransferase 5

The *N*-acetylgalactosaminyltransferase 5 gene with three unique intron positions did not show any significant relationship with other transferases and was classified as Id subfamily [11]. GalNAcT5 expression was found to be highly tissue-specific and catalyzing the glycosylation of a particular subset of peptides [14, 58]. Moreover, GalNAcT5 was localized in gastric epithelial cancer cells, and strong expression of GalNAcT5 correlated with well-differentiated gastric carcinoma whereas moderate or poor expression correlated with less differentiated carcinomas. In the same study, a closer follow-up and aggressive therapeutic treatment was advised for the gastric cancer patients with low intratumoral GalNAcT5 expression [59]. In breast cancers, presence of two missense mutations (p.E507D and p.L692F in the catalytic domain) of *GALNT5* appeared to reduce the transferase activity of GalNAcT5 [60]. However, the sequence analyses of *GALNT5* in a group of thirty microsatellite-stable colon cancer cell lines failed to confirm the presence of these previously identified somaticmutations [60, 61].

Cytogenetic data of hepatoblastoma have revealed that up-regulation of *GALNT5*, *DAPL1*, *ERMN*, *SCN1A* and *SCN3A* plays a major role in the development and progression of the disease [62]. However, the post-translational modifications regulated by GalNAcT5 in hepatoblastoma cells remain undefined, as the mechanism of action of *DAPL1*, *ERMN*, *GALNT5*, *SCN1A* and *SCN3A* genes.

### *N*-acetylgalactosaminyltransferase 6

The *GALNT6* gene is structurally identical to *GALNT3*, and grouped in the Ic subfamily [11]. Numerous studies based on different experimental approaches have suggested a correlation between expression of GalNAcT6 and T3 with tumor differentiation [41]. However, only GalNAcT6 expression was predicted as a prognostic factor whereas GalNAcT3 was not [40, 63].

Most studies on GalNAcT6 have shown its pivotal role in breast cancers [63-65]. During human breast carcinogenesis, strong expression of GalNAcT6 in ductal carcinoma in situ (DCIS) was considered an early event leading to aberrant mucin O-glycosylation [63]. Moreover, the selective expression of GalNAcT6 in myoepithelial cells in some breast cancer patients was associated with angiogenesis and invasiveness [63].

In breast cancer cells, GalNAcT6 may cause aberrant glycosylation of Muc1, thus mediating mammary carcinogenesis by up-regulating cell adhesion molecules *β*-catenin and E-cad [66]. The level of GalNAcT6 was found significantly higher in breast cancer cells as compared to normal or benign mammary cells [64]. Moreover, fibronectin, an important *in vivo* substrate of GalNAcT6/GalNAcT3, was stabilized by GalNAcT6-induced O-glycosylation and did not undergo degradation after endocytosis. Overexpression of *GALNT6* and O-glycosylation of fibronectin increased the transformation of mammary epithelial cells and abrogated their proliferative behavior *in vivo* [65]. This GalNAcT6-fibronectin pathway may therefore matter in breast cancer development and progression, but tumor heterogeneity and the

In response to cigarette smoke, degradation of E-cadherin (E-cad) in lung cancer cells resulted from shedding of a 400 kDa Muc1-N isoform, leading to Muc1-C glycosylation and complex formation with p120ctn through bridging of Src/Muc1-C/galectin-3/EGFR signalosomes. This smoke-induced Muc1-C glycosylation and Muc1-C/p120ctn interaction was suppressed with *GALNT6* shRNA, thereby inhibiting E-cad degradation— a major hallmark of epithelial-mesenchymal transition (EMT)— by blocking the smoke-induced Muc1-N shedding and maintaining cellular polarity [67].

### *N*-acetylgalactosaminyltransferase 7

GalNAcT7 is a peptide-preferring transferase that was grouped with *GALNT10* and *GALNT17* in the IIb subfamily [11].

Overexpression of *GALNT7* is associated with carcinogenesis and metastasis in laryngeal squamous cell carcinoma (LSCC) [1], hepatocellular carcinoma [68], cervical cancer [2], superficial spreading melanoma (SSM) and nodular melanoma (NM) [69]. Through reduced expression of *GALNT7*, miR-34a and miR-34c are tumor suppressors in laryngeal squamous carcinoma cell (LSCC), thereby serving as novel potential markers for LSCC therapy [1]. Likewise, down-regulation of miR-214 and up-regulation of its target gene *GALNT7* have been documented in cervical cancer cells but not in adjacent normal tissues [2]. Superficial spreading melanoma (SSM) and nodular melanoma (NM characteristically express the eight genes *GALNT7*, *DIS3*, *FGFR1OP*, *G3BP2*, *MTAP*, *SEC23IP*, *USO1*, and *ZNF668* [69].

Up-regulation of miR-30d or silencing of *GALNT7* has significant effects on the O-glycosylation of melanomas. During melanoma progression, the expression of *GALNT7* is decreased by miR-30b/30d, which promotes cell invasion and immunosuppression by altering the O-glycosylation patterns of membrane proteins interacting with the ECM and cells of the tumor environment [20]. The suppression of *GALNT7* with upregulation of miR-30d increases the secretion of IL-10, providing a possible mechanistic explanation for the immunosuppressive behavior of melanomas [20].

*GALNT7PLA2SIAT8B*

*ACVR2A, AJAP1, CA12, CDK12, FAM38A, GALNT7, LMO3, MTA1, SLC19A1, SLC43A3, ZNF493* *CA12GALNT7LMO3SLC43A3,* eregulated in post-Chernobyl thyroid cancer [72].

### *N*-acetylgalactosaminyltransferase 8

*GALNT8* was classified in the Ie subfamily with *GALNT9*, *GALNT18* and *GALNT19* [11], however, in another classification system, the *GALNT8*, together with *GALNT9*, *GALNT17* and *GALNT18*, were defined as the Y-subfamily [73].

Through large-scale association analysis including over a thousand Japanese patients with chronic hepatitis C (CHC), *GALNT8* variants were found associated with the outcome of interferon therapy [74]. In another report on a Pakistani population, no such correlation was apparent for the *GALNT8* variant rs10849138 [75]. However, further large scale population-based studies are needed to assess the role of rs10849138 in this Pakistani population.

### *N*-acetylgalactosaminyltransferase 9

The *N*-acetylgalactosaminyltransferase 9 gene (*GALNT9*) belongs to both Y-subfamily [73] and Ie subfamily of *GALNTs* [11].

Among thirty-three oncogenes, fourteen genes including *GALNT9* have been characterized with a novel hotspot mutation in colorectal cancer (CRC) [76]. Although the molecular mechanism involving *GALNT9* and other factors in low-risk neuroblastoma patients remain largely unknown, the *GALNT9* has been validated as a prognostic marker that may be helpful to guide therapy in low-risk neuroblastoma patients [77]. Lastly, a recent bioinformatic screen suggested that *GALNT9,* together with *CCDC8* (involved in microtubule regulation) and *BNC1* (transcription factor), may support brain metastatisation of breast carcinoma [78].

### *N*-acetylgalactosaminyltransferase 10

GalNAcT10 is a glycopeptide-preferring transferase exhibiting unique sequence-recognition properties and stands out among other glycopeptide-preferring transferases [11, 13].

In the liver, *GALNT10* promotes carcinogenesis and was recognized as a bona fide target for miR-122, a liver-specific mammalian miRNA. Moreover, in hepatitis B virus (HBV)-associated hepatocellular carcinoma (HCC), hepatocyte nuclear factor 4α (Hnf4α) was found to activate *miR-122* gene transcription. The reduced expression of miR-122 might facilitate GalNAcT10 expression to promote proliferation and apoptosis resistance of HCC in a glycosyltransferase-dependent manner [79]. By modifying the O-glycosylation of EGFR and subsequent phosphorylation of AKT, the GalNAcT10 increased EGFR signaling, the development of HBV-associated HCC proliferation and resistance to apoptosis. Actually, GalNAcT10 promoted HCC by aberrantly glycosylating Muc1 and several other glycoproteins and surface molecules, in addition to EGF. Activated GALNT10 therefore, facilitates HCC tumor growth in HBV-infected cells, but *GALNT10* activity appears to be decreased under the influence of Hnf4α and miR-122 [79].

The involvement of *PSG11* and *GALNT10,* together with *SLC2A2, SLC17A, CD53, THBS2, LCT,* and *GYPA* was suggested in O-glycoprotein synthesis [80]. The coactivation of these genes suggest early tumor growth and could provide useful information for risk assessment in ovarian cancer patients. Recent GWAS studies have shown association of different SNPs at or nearby *GALNT10* with high body mass index (BMI), predominantly in African and European populations, suggesting a distinct role for *GALNT10* in adiposity within different ethnic populations [81-83].

The biological consequences of organ-specific glycosylation by GalNAcT10 in the stomach, small intestine, liver, pancreas, ovary, spleen, and central nervous system (CNS) are still to be explored [84, 85]. The same holds true for the molecular mechanisms underlying the relationship between adiposity and *GALNT10* expression.

### *N*-acetylgalactosaminyltransferase 11

The expression of the Tn and sialyl-Tn antigens usually reflects incomplete/aberrant O-glycosylation and are a hallmark of different types of cancers, frequently associated with poor prognosis. In a recent report on chronic lymphocytic leukemia (CLL), a low density of Tn residues and higher expression of *GALNT11* was observed in B-CLL cells and healthy T-cells suggesting that GalNAcT11 contributes to B- and T lymphocyte differentiation and transformation [36]. In another context, meta-analyses of GWAS for 63,558 individuals of European origin correlated *GALNT11*, *CDH23* and *UMOD* with the rapid decline of kidney function [86].

### *N*-acetylgalactosaminyltransferase 12

Unfolded protein response (UPR) pathways are activated by aberrant glycosylation that induces ER (endoplasmic reticulum) stress [87]. UPR restores ER homeostasis, but prolonged ER stress causes apoptosis. Deficiency in Mixed Lineage Leukemia 1 (MLL1: a mammalian histone H3K4 methyltransferase) enhanced UPR and apoptosis caused by the N-glycosylation inhibitor tunicamycin (TM), and the direct binding of MLL1 to the promoters of *GALNT12, H6PDUGP2* activated the expression of these genes. When all three genes were knocked-down, TM-induced apoptosis was enhanced. However, transfecting the *GALNT12, H6PDUGP2* enhanced glycosylation and maturation of Lamp2 (lysosome-associated membrane protein 2). Therefore, faulty glycosylation of Lamp2 by transferases (e.g. GalNAcT12) may lead to cancer and developmental defects [86, 87].

In two-hundred and forty-four follicular lymphoma (FL) cases recognized during a population-based case-control study of non-Hodgkin lymphoma (NHL), five genes (*GALNT12*,*BMP7*, *DUSP2*,*GADD45B*, and*ADAM17*) were found associated with the overall survival of FL and control of B-cell activity. *mutated GALNT12* was associated with aberrant glycosylation, but inherited differences in growth-regulatory pathways of immune cells may have influenced the phenotype and progression of FL [88]. In a colon cancer study, eight mutations inactivated the normal function of the GalNAcT12 to variable degrees (reduction in enzyme activity: p.R382H, 1%; p.T491M, 2%; p.R373H, 5%; and p.R279W, 7% and p.D303N, 37%), leading to variable phenotypes of colon and breast carcinoma [60]. Although breast cancer is commonly regulated by GalNAcT4 and GalNAcT12, more is needed to assess the underlying mechanism associating the two enzymes in the same cancer cell.

In the majority of hereditary colorectal cancer (CRC) families, *GALNT12* variants were characterized as highly penetrant variants that influence the pathogenesis of CRC [89, 90], but little is known regarding the genotypic and allelic frequencies of *GALNT12* in different racial/ethnic groups. Moreover, in addition to *GALNT12*, different other genes (*ZNF367, HABP4,* and *GABBR2*) are also associated with CRC susceptibility.

### *N*-acetylgalactosaminyltransferase 13

Both *GALNT1* and *GALNT13* were found almost 90% identical in structure, substrate specificity and kinetic properties, and grouped in subclass Ia [11]. However, GalNAcT13 showed a higher specificity for Muc5Ac and Muc7 than GalNAcT1 [18].

*GALNT13*, like *GALNT1*, is highly expressed in all neuroblastoma (NB) cells, but not in glioblastoma cells [18]. As the bone marrow (BM) is the preferential site for NB dissemination, BM molecular analysis of human neuroblastoma patients has shown high expression of *GALNT13,* which was identified as a new indicator for disseminated neuroblasts in BM of NB patients. GalNAcT13, unlike GalNAcT1, may be a major glycosyltransferase in the biosynthesis of *O*-glycans in the brain and contribute to the formation of a triplet Tn epitope on peptides on cell-surface transmembrane heparan sulfate proteoglycans (syndecan-3) in neurons and Schwann cells [91]. However, the role of the triplet *O*-glycan formation on syndecan-3, if any in brain cancers, is not known.

In an immunohistochemical study, inactivation of *GALNT13* showed a remarkable decrease in expression of the Tn antigen in cerebellum, suggesting the implication of the GalNAcT13 enzyme in neuronal function [18].

### *N*-acetylgalactosaminyltransferase 14

Overexpression of *GALNT14,* which initiates the O-glycosylation of mucin substrates Muc2, Muc5Ac, Muc7 and Muc13, activates the invasion and migration of breast cancer cells by up-regulating MMP-2VEGF, TGF-β, *N*-cadherin, vimentin and down-regulating E-cad [92]. Of the two single nucleotide polymorphisms rs9679162 and rs6752303 flanking *GALNT14*, the TT genotype of rs9679162 was strongly associated with the non-viral etiology of HCC [93]. In another similar study on advanced HCC patients treated with 5-fluorouracil, mitoxantrone and cisplatin chemotherapy, the *GALN14* genotype rs9679162 was found to be a reliable marker for a positive therapeutic outcome [86]. The *GALNT14* genotype and α-fetoprotein (AFP) levels were identified as pre-therapeutic markers for a favorable response in HCC [86]. In another study on ovarian cancer, suppression of cell migration and altered cellular morphology were found to result from the knockdown of *GALNT14* by small interfering RNA. In the same report, interleukin-8 (IL-8) had no significant effect on the function of GalNAcT14 and the tumor-associated Tn antigen [94]. However, co-expression of GalNAcT14 and transmembrane mucin 13 (Muc13) in ovarian cancer tissues, but not in normal tissues, suggested a contribution of GalNAcT14 to ovarian carcinogenesis through aberrant glycosylation of Muc13. The exact molecular mechanism underlying the Muc13-GalNAcT14-ERK1/2 pathway remains however undefined [94].

*GALNT14GALNT14GALNT14*

In CRC cell lines, dulanermin sensitivity was correlated with GalNAcT14 activity on Muc1 substrate and the fucosyltransferase enzymes FUT3 and FUT6 [95, 97]. In patients with advanced hepatocellular carcinoma, linkage of the germline SNP marker rs9679162 with *GALNT14* could be correlated with the objective response to the first course of 5-fluorouracil, mitoxantrone and cisplatin (FMP) chemotherapy [98]. However, the role of rs9679162 in the FMP response and its influence on the alternative splicing of *GALNT14* and other *GALNTs* [98] remain unknown.

### *N*-acetylgalactosaminyltransferase 15 (*GALNT15*) to *N*-acetylgalactosaminyltransferase 20

Similar to *GALNT5*, *GALNT15* did not show any significant relationship with other *GALNT* family members and was grouped in the Ig subfamily [11]. After genotyping of CRC cases and controls in the EPICOLON consortium, four genetic variants, rs2102302 in *GALNTL2*, rs3803185 in *ARL11*, rs698 in *ADH1C* and rs1800795 in *IL6*, were found with potential association to CRC risk factor [99].

*GALNT16* shares the same intron numbers (with minor variations in introns positions) with *GALNT2* and *GALNT14* and the three genes were grouped together as Ib subfamily [11]. The GalNAcT18 (encoded by *GALNT18*/*GALNTL4*) was classified as Ie subfamily with *GALNT8*, *GALNT9* and *GALNT19* [11, 100, 101], but in another classification system, the *GALNT8*, *GALNT9*, *GALNT17* and *GALNT18* were defined as the Y-subfamily. All Y-subfamily member enzymes fail to glycosylate classical substrates because of an altered UDP-GalNAc-binding pocket in LDX(5)YGGENXE. Nonetheless, the expression profile of GalNAcT18 in the endoplasmic reticulum of lung carcinoma cells and the observed enhancement of catalytic activity of both GalNAcT2 and GalNAcT10 in the presence of GalNAcT18 suggests that GalNAcT18 could be a chaperone-like protein [73].

*N*-acetylgalactosaminyltransferase-like protein 5 (*GALNTL5*) is the only GalNAcT family member of which lacks the lectin domain, and has been called GalNAcT19 or GalNAcT20 in the literature (Refseq accession no.: NP_660335.2) [102]. However, no clinical evidence has been reported for any involvement of *GALNTL5* in cancer.

## CONCLUSIONS AND PERSPECTIVES

The GalNAcTs family is involved to variable degrees in carcinomas, leukemias, lymphomas and central nervous system cancers, and several non-neoplastic diseases and developmental defects. Developmental pathologies involving cardiac valves and submandibular gland generation also depend on the timely and efficient participation of *N*-acetylgalactosaminyltransferases during ontogeny. This critical role of the sugar transferases catalyzing O-glycosylation of cell-surface proteins (mucins and surface receptors like DR4/DR5, IGF-lR, MET, and EGFR) and ECM proteins (fibronectin) points to the fundamental involvement of glycoproteins in interactions of epithelial and hemopoietic cells with the ECM and small proteins like cytokines, growth factors and hormones. Therefore, proliferative activities and metastatic behaviors may all depend upon O-glycosylated cell-surface receptors and cell-cell interaction proteins. Lastly, metabolic diseases such as diabetes and calcinosis were shown to display genetic alterations of *GALNT2* and *GALNT3,* respectively, alterations reflected by inappropriate insulin responses and calcium deposition in the case of calcinosis [103-106].

Site-specific GalNAc-type O-glycosylation is also a major regulator of proprotein convertase processing, which generates a variety of active extracellular agonists like the endocrine FGF23 [107]. It has been estimated that the number of proteins generated in this manner well exceeds 3000, including cytokines and hormones, but also proteases and receptors. The range of effects generated by deficient preprocessed proteins is expected to be very large, and will certainly be part of developmental defects and neoplastic phenotypes.

**Figure 2:**
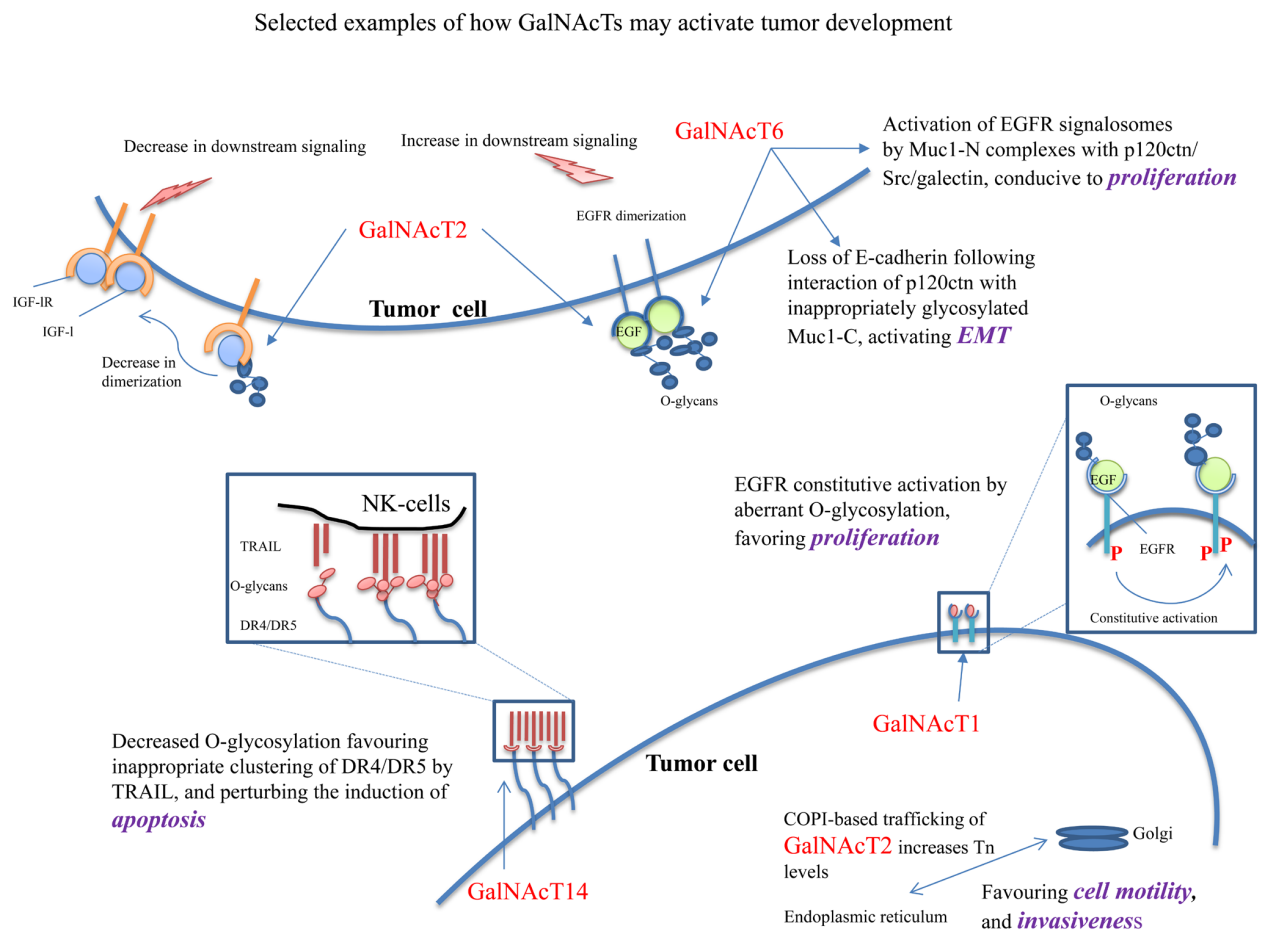
Selected examples of putative mechanisms whereby GalNAcTs could support the neoplastic phenotype 1) Constitutive activation of IGFR-1 dimers, under O-glycosylated by GalNAcT2, 2) constitutive activation of EGFR dimers, under O-glycosylation by GalNAcT2 and T6), 3) loss of E-cadherin following interaction with p120-catenin with inappropriately glycosylated Muc1-C, by GalNAcT6, activating EMT, 4) activation of EGFR signalosomes of MuC-1 complexes with p120ctn/Src/galectin, by GalNAcT6, leading to proliferation, 5) COPI-based trafficking of GalNAcT2 increases the amounts of Tn antigen in the ER, leading to increased cell motility and invasiveness, and 6) decreased O-glycosylation of DR4/DR5 suppresses the clustering of TRAIL, perturbing the induction of apoptosis.

The wide spectrum of biological consequences that could result from altered *N*-acetylgalactosaminyltransferase biological activities (see Figure 1) mostly fits the contexts of 1) cellular interactions with the ECM, as exemplified by neoplastic cells interacting with the peri-tumoral context during invasion, or epithelial cells contacting their basement membrane, 2) abnormal responses of abnormally O-glycosylated cell-surface receptors to ECM ligands, insulin and cytokines, as expected to happen in epithelial cells during oncogenesis, insulin resistance, inappropriate responses to growth factors and resistance to apoptosis-inducing ligands, 3) alteration of vesicle trafficking genes, as it has been suggested in 17β-estradiol-resistance in breast cancer cells, 4) altered mucin-type O-glycosylation of the cellular glycocalyx, as described in regulation of various carcinomas, and 5) COPI-based translocation of GalNAcTs to the endoplasmic reticulum, as it has been demonstrated in driving motility and invasiveness of tumor cells (Figure 2).

In particular, the frequent occurrence of modulation by GalNAcTs of transmembrane receptors points to the selective influence of O-glycosylation on receptor dimerization and activation of their intracytoplasmic tyrosine kinases (Figure 2). This applies to insulin and insulin-like receptors, apoptosis receptors (DR4/5), MET and TGF-β receptors, in normal and neoplastic cells, and most selectively to growth factor receptors (EGFRs & IGF-lR) in neoplastic cells. Mechanistically, inappropriate O-glycosylation of tyrosine kinase receptors in cancer cells may inactivate them by preventing their dimerization [27] or abnormally favor their dimerization [34, 108] and make them constitutively active (Figure 2). Both outcomes may take place in normal cells and lead to insufficient or excessive proliferation during embryogenesis and result in growth defects. In cancer cells, however, constitutive proliferation is a hallmark of neoplastic behavior [109]. It follows that carcinoma cells may express a wide variety of tyrosine kinases transmembrane proteins which may [27, 33, 34, 108] or may not be appropriately O-glycosylated by the available transferases. Given this unpredictability in oncogenic driver expression, as for instance in NSCLC [110], gastric adenocarcinoma and HCC, altered O-glycosylation of the oncogenic driver may sustain hyperproliferation in many different ways [27, 33].

As highlighted in Figure 1, genetic alterations in *GALNTs* are almost never isolated events, but always associated with other genetic alterations, as shown by GWAS and non-GWAS studies. To find out how the expression of *GALNTs* products and other altered genes concurrently contribute to the pathologies ascribed to *GALNTs'* alterations is a most interesting challenge.

It is expected that further GWAS and other linkage studies, and especially the availability of specific antibodies to individual transferases and biochemical characterization of the transferases themselves, will provide not only the means of ascribing individual transferase to specific clinical contexts, but also to identify those transferases that could be targeted therapeutically.
